# Evaluation of the Clinical Utility of Circulating Tumor DNA in the Management of Patients With Nonmetastatic Head and Neck Cancer: Protocol for a Prospective Bicentric Study (CIRVAD)

**DOI:** 10.2196/89973

**Published:** 2026-11-05

**Authors:** Romina Mastronicola, Maëlys Bresson, Giacomo Gravante, Sophie Cortese, Emilie Beulque, Fadila Chergui, Alexandre Harlé, Gilles Dolivet

**Affiliations:** 1 Head and Neck Department, Institut de Cancérologie de Lorraine, 6 avenue Bourgogne, Vandoeuvre les Nancy, Grand Est, France, 33 782467069; 2 Centre de Recherche en Automatique de Nancy, Vandoeuvre les Nancy, Grand Est, France

**Keywords:** circulating tumor DNA, ctDNA, upper aerodigestive tract cancer, surgery, plasma analysis, head and neck

## Abstract

**Background:**

Human papillomavirus (HPV)–negative head and neck cancers, often linked to tobacco and alcohol exposure, have a poor prognosis and high recurrence rates. Circulating tumor DNA (ctDNA) is a promising biomarker for detecting minimal residual disease and monitoring treatment response. Recent studies show that ctDNA can detect recurrence earlier than clinical or radiological methods. Prospective trials are needed to validate its clinical utility.

**Objective:**

The primary objective is to assess postoperative ctDNA as a predictor of 28-month progression-free survival. Secondary objectives include evaluating ctDNA levels before and after treatment as predictors of recurrence, studying ctDNA dynamics during follow-up in relation to survival, and exploring their association with tumor characteristics and surgical outcomes.

**Methods:**

This prospective, bicentric study evaluates ctDNA in patients undergoing surgery for head and neck squamous cell carcinoma. Plasma ctDNA concentrations will be measured quantitatively in ng/mL before and 1 to 2 weeks after surgery, with or without adjuvant radiotherapy or chemotherapy. Progression-free survival is defined as the time from surgery to disease progression or death, and recurrence-free survival as the time to clinically confirmed recurrence. Overall and disease-specific survival will also be assessed. Changes in ctDNA levels over follow-up and clinical characteristics will be compared between patients with and without progression at 28 months. Absolute and relative variations in ctDNA preoperatively and postoperatively will be described, and surgical quality will be evaluated according to resection margins (R0, R1, R2).

**Results:**

As of August 2026, study preparation, including protocol development and methodological validation, has been completed. The study is currently entering the implementation phase, with regulatory procedures, funding applications, and site initiation activities underway. No participants have been recruited at the time of manuscript submission, as recruitment has not yet begun. Recruitment is expected to start following regulatory approval and funding acquisition. The funding status remains pending as of August 2026, with funding applications underway. Participant recruitment and data collection are projected to take approximately 24 months, followed by a 28-month follow-up period. Data analysis has not yet started and will be conducted after completion of the follow-up period. The first study results are therefore expected to become available after the completion of data collection and follow-up, although a precise publication timeline (season/year) cannot yet be determined.

**Conclusions:**

This study aims to provide key data on the potential of ctDNA to detect early recurrence in HPV-negative cancers and could inform personalized patient monitoring and management.

## Introduction

### Background

Head and neck squamous cell carcinomas are among the most common cancers worldwide (sixth) and rank as the eighth leading cause of global mortality [1]. In 60% of cases, the cancer is locally advanced, with a recurrence rate of 50%, which is most often incurable [2]. Tobacco and alcohol consumption are predisposing factors, as are infections with human papillomavirus (HPV) and Epstein-Barr virus. Hereditary genetic factors also predispose individuals to these cancers [3].

HPV-negative cancers, resulting from tobacco and alcohol exposure, are characterized by genetic instability and a mutation profile distinct from HPV-positive cancers. HPV-negative cancers are resistant to chemotherapy and radiotherapy and have a poorer prognosis than HPV-positive tumors. Indeed, patients with HPV-negative tumors have a lower 3-year overall survival compared with HPV-positive patients (3-year overall survival rates of 57.1% vs 82.4%). Three-year progression-free survival (PFS) rates are 43.4% and 73.7% in the HPV-negative and HPV-positive groups, respectively [3].

HPV-negative tumors also show a higher tendency to recur. In a prospective study of 124 patients with advanced oropharyngeal tumors (86% stage IV), HPV-negative patients were 3.5 times more likely to develop disease recurrence (hazard ratio 3.5, 95% CI 1.6-7.9; *P*=.002) than HPV-positive patients. Three-year recurrence rates were approximately 54% for HPV-negative patients compared with 29% for HPV-positive patients [4].

Most head and neck cancers are diagnosed at an advanced stage (70.3% stage III/IV). Early recurrence after treatment is difficult to detect with current imaging modalities and tumor markers due to the anatomical location and previous treatments of the primary tumor (radiotherapy, surgery, and reconstruction). In addition, the spatial, epidemiological, genetic, and epigenetic heterogeneity of these cancers further complicates the prediction of recurrence risk [5].

Currently, no effective biomarker exists to assess treatment response or early recurrence in HPV-negative patients. Circulating tumor DNA (ctDNA) may represent a promising biomarker for detecting minimal residual disease and monitoring treatment response. ctDNA consists of small DNA fragments derived from the tumor, 150 to 200 base pairs in length, that are present in the blood of patients with cancer [6]. This DNA is released into the bloodstream following apoptosis or necrosis of tumor cells and carries somatic mutations from primary and secondary tumors. The presence of ctDNA was first demonstrated in 1994 by Sorenson in patients with pancreatic carcinoma, showing that the same Kirsten rat sarcoma viral oncogene homolog gene mutation was present in both the tumor and circulating DNA [7].

Liquid biopsy, a technique developed in oncology over the past 50 years, allows the analysis of ctDNA in patients with cancer. This method is noninvasive, relatively inexpensive, and can provide multiple pieces of information, including genetic and epigenetic profiles, detection of minimal residual disease, and monitoring of treatment response [8-11]. Therefore, ctDNA detection could become a potential biomarker for HPV-negative head and neck cancers.

Longitudinal monitoring using Epstein-Barr virus and HPV DNA (E6/E7 oncogene segments) has been extensively studied, in contrast to the evaluation of ctDNA in HPV-negative patients [12-14]. A 2018 study of 7 HPV-positive and HPV-negative patients aimed to evaluate ctDNA as a biomarker for detecting recurrence or treatment response after surgery or chemoradiotherapy. ctDNA was detectable before treatment in all patients with stage IV cancer. Two patients with local recurrence had detectable ctDNA prior to clinical relapse, whereas no mutations were detected in 2 patients who developed distant metastases before clinical recurrence [15].

Several studies investigated the correlation between ctDNA, treatment response, and recurrence in 26 patients with head and neck squamous cell carcinoma (22 HPV-negative and 4 HPV-positive). Tumor mutations were analyzed along with peripheral blood mononuclear cells, and each patient’s mutations were tracked in the blood. Mutated ctDNA was detectable before treatment initiation in 15 of 18 analyzed patients (6 were excluded due to probe design failure). The 3 patients without detectable ctDNA before treatment included 2 with stage I tumors and 1 with external auditory canal carcinoma.

After curative treatment, ctDNA was detected in all patients with local recurrence or distant metastases (7/7), whereas the 11 patients with undetectable ctDNA remained alive and recurrence-free during follow-up.

This study had several limitations, including heterogeneous patient characteristics (stages I-IV, HPV-negative and HPV-positive) and curative treatments. Follow-up was also heterogeneous, with a median of 563 (range 96‐1033) days [16].

A recent prospective single-center study in 2022 included 17 patients with stage III or IVb HPV-negative squamous cell carcinoma of the oral cavity, pharynx, or larynx. All patients received primary curative surgery. The study primarily aimed to determine whether postoperative ctDNA detection could serve as a biomarker for minimal residual disease and recurrence. Patients were followed for a median of 12.2 (range 9.6‐17.5) months, and preoperative ctDNA was detected in all patients. During longitudinal follow-up, ctDNA was detected before clinical progression in all cases with clinically confirmed recurrence (5/17 patients, 29%), with lead times of 3.5 to 8.3 months. Among the 12 patients without evidence of recurrence at the last follow-up, all ctDNA samples were negative [17].

Finally, a clinical trial (NCT03712566) included 55 patients (37 HPV-negative and 15 HPV-positive) with recurrent or metastatic disease to assess longitudinal ctDNA kinetics during treatment. ctDNA was detected at baseline in 94.3% of patients. Early changes in ctDNA dynamics after 1 treatment cycle better predicted both progression-free and overall survival than baseline ctDNA levels. Moreover, a decrease in ctDNA from baseline to postcycle 1 identified patients with longer overall survival despite radiological progression [18].

These studies demonstrate that longitudinal ctDNA monitoring in patients with head and neck cancer is feasible and could serve as a useful biomarker for early recurrence detection, providing clinicians with additional guidance in therapeutic decision-making. However, given the small number of patients analyzed, heterogeneous populations, varied treatments, and inconsistent follow-up, larger prospective studies are required before this approach can be implemented in routine clinical practice.

### Need for This Study

This study is justified by the need to develop more precise tools for the early prediction of recurrence in patients with ENT (ear, nose, throat) cancers, particularly those who are HPV-negative, a population for which surveillance and treatment options are currently limited. By evaluating ctDNA concentrations postoperatively and during follow-up, we may be able to predict recurrence before it is detectable by conventional clinical or imaging methods. This innovative approach aims to identify a ctDNA threshold indicative of an increased risk of recurrence, enabling earlier and tailored therapeutic interventions. Given the limited number of studies conducted on HPV-negative patients, who have a poorer prognosis compared with HPV-positive patients, it is essential to focus efforts on this specific cohort. Furthermore, the ability to more rapidly predict recurrence using ctDNA could not only improve patient monitoring and management but also allow for earlier therapeutic adjustments, such as considering a second surgery or modifying treatment.

There will be no direct benefit for patients included in this study. Nevertheless, the CIRVAD study will contribute to advancing scientific knowledge on the correlation between ctDNA concentration and recurrence and provide additional insights into the emergence and monitoring of mutations in patients with HPV-negative ENT cancers undergoing treatment.

If positive results are obtained, this study could help establish an additional postoperative monitoring method (ctDNA concentration) that is low-cost, noninvasive, and minimally burdensome for patients with head and neck cancers. The study may also allow surgeons to assess the quality of surgical procedures. These findings could ultimately enable personalized follow-up to identify recurrence risk early through a simple blood test and optimize therapeutic strategies.

The risks associated with participation in the CIRVAD protocol are minimal, as patient management (treatments and examinations) follows standard care for patients with this pathology. Individual risks related to venipuncture are minimal and comparable to those associated with routine blood sampling. Patients may occasionally experience mild pain or bruising. Rarely, fainting, small blood clots, or infection may occur at the puncture site. These risks are negligible, as they are mitigated by proper skin asepsis and the expertise of experienced personnel performing the procedure.

### Objectives

A 28-month follow-up period was selected because most recurrences of head and neck squamous cell carcinoma occur within the first 2 years after curative treatment, corresponding to the period of highest recurrence risk and the most intensive postoperative surveillance recommended by international guidelines. Extending follow-up to 28 months accommodates study logistics while ensuring capture of most clinically relevant recurrence events. This timeframe is therefore appropriate for evaluating the ability of early postoperative and longitudinal tumor-informed ctDNA analysis to detect molecular residual disease and predict recurrence [3,4].

Secondary objectives include evaluating plasma ctDNA concentrations before surgery, in the early postoperative period, and during follow-up as predictive factors for clinical recurrence following therapy; assessing quantitative ctDNA concentrations and their longitudinal evolution as predictors of progression-free, overall, and disease-specific survival; describing the clinical characteristics of patients and the evolution of quantitative ctDNA concentrations during follow-up in patients with and without progression throughout the 28-month follow-up period; and describing the variation in quantitative ctDNA concentrations between the preoperative and postoperative periods and analyzing their relationship with tumor size and surgical quality. The study will also determine the proportion of patients with detectable preoperative plasma ctDNA and evaluate its feasibility as a biomarker for longitudinal disease monitoring.

## Methods

### Study Design and Outcome Measures

This is a prospective, bicentric study. This protocol was developed in accordance with the SPIRIT (Standard Protocol Items: Recommendations for Interventional Trials) 2013 Statement [19].

Plasma ctDNA samples will be collected before surgery, in the early postoperative period (1‐2 weeks after surgery), and longitudinally during scheduled follow-up visits throughout the 28-month follow-up period. For patients receiving adjuvant radiotherapy and/or chemotherapy, ctDNA analysis will be performed after completion of adjuvant treatment according to the study schedule. Plasma concentrations of ctDNA will be quantitatively expressed in ng/mL of plasma.

PFS is defined as the time from surgery to disease progression or death (event: local or regional progression, distant metastases, or death from any cause).

Plasma ctDNA concentrations obtained at each time point will be used to evaluate the association between molecular residual disease and clinical outcomes, including recurrence, PFS, overall survival, and disease-specific survival.

Time to recurrence is defined as the interval between surgery and clinically confirmed recurrence (event: local or regional recurrence, distant metastases). Overall survival is defined as the time from surgery to death from any cause, and disease-specific survival as the time from surgery to cancer-related death.

The evolution of quantitative ctDNA concentrations during follow-up and the clinical characteristics of the study population will be described and compared between patients with and without progression throughout the 28-month follow-up period. Relative and absolute variations in plasma ctDNA concentrations between the preoperative and postoperative periods will be described. The quality of the surgical procedure will be evaluated according to resection margin classification (R0, R1, and R2).

### Ethical Considerations

#### Regulatory Requirements

The study will be conducted in accordance with the ethical principles of the latest version of the Declaration of Helsinki [20]; Good Clinical Practice guidelines dated November 24, 2006, as defined by the International Council for Harmonisation (ICH-E6) [21]; the French Data Protection Act number 78‐17 of January 6, 1978, as amended by Law number 2004‐801 of August 6, 2004, relating to the protection of individuals with regard to the processing of personal data [22]; bioethics law number 2004‐800 of August 6, 2004 [23]; decree number 2016‐1537 of November 16, 2016, concerning research involving human subjects [24]; the Order of May 7, 2017, establishing the list of research studies mentioned in Article L.1121‐1, paragraph 2, of the Public Health Code [25]; and the Order of May 9, 2017, modifying certain regulatory provisions relating to research involving human subjects [26].

#### Ethics Committee, Competent Authority, CNIL (National Data Privacy Commission), and Insurance

The study will be submitted to the appropriate French Ethics Committee (Comité de Protection des Personnes, CPP) and to the competent regulatory authorities before the start of patient recruitment. Patient inclusion will not begin until all required regulatory approvals have been obtained.

#### Patient Information and Informed Consent

Before conducting minimal-risk interventional research on a person, their free, informed, and explicit consent (written or oral) must be obtained after exhaustive information is provided by the investigator during a consultation and after a sufficient reflection period. The sponsor may request that the participant agree, at the time of giving informed consent, that their data be used in future research exclusively for scientific purposes. The participant may withdraw consent for this future use or exercise their right to object at any time.

Similarly, if a protocol amendment requires revising the information forms provided to participants and necessitates obtaining new consent, the submission to the relevant ethics committee must include a description of the procedures planned to collect this new consent.

For studies aiming to perform genomic or proteomic analyses, the information form must specify the type of research to be conducted. The use of these samples for medical or scientific purposes other than those for which they were collected is allowed unless the participant objects after being properly informed. Exceptions to the obligation to inform may occur when it is impossible to contact the person concerned or when one of the advisory ethics committees mentioned in Article L.1123-1, consulted by the research sponsor, does not deem such information necessary [27].

Personal information must be kept in the medical file of the participating physician and treated as strictly confidential, but it must be accessible for review by the competent authorities and duly authorized personnel.

According to Article 1122-1-1, if a participant withdraws their consent, this withdrawal does not affect activities already carried out or the use of data obtained based on the informed consent provided before withdrawal [28].

### Study Population

#### Study Population and Inclusion/Exclusion Criteria

The study will be presented to treatment-naïve patients with nonmetastatic HPV-negative squamous cell carcinoma of the upper aerodigestive tract during an ENT consultation or day hospitalization (Textbox 1).

Textbox 1. **Inclusion and exclusion criteria.**

**Inclusion criteria**

- Patients aged 18 years or older.
- Patients with histologically confirmed squamous cell carcinoma of the upper aerodigestive tract (nasal cavity, sinuses, oral cavity, pharynx, larynx, or hypopharynx) who are treatment-naïve for this cancer.
- Patients with nonmetastatic and resectable disease at diagnosis.
- Patients who are human papillomavirus (HPV) negative (p16-negative).
- Patients requiring surgical intervention for cancer treatment.
- Patients able and willing to comply with all study procedures according to the protocol.
- Patients who have understood, signed, and dated the informed consent form provided at inclusion.
- Patients affiliated with the social security system.

**Exclusion criteria**

- Minors.
- Patients who are HPV-positive (p16-positive).
- Patients previously treated for head and neck cancer.
- Patients receiving immunotherapy and/or targeted therapy.
- Patients with another cancer in the past 5 years (other than head and neck cancer).
- Patients with metastatic disease.
- Pregnant or breastfeeding women, or women of childbearing potential not using contraception.
- Individuals deprived of liberty or under guardianship (including curatorship).

#### Sample Size Calculation

This is a pilot study aiming to include 60 patients with stage III or IV HPV-negative squamous cell carcinoma of the upper aerodigestive tract to evaluate whether postoperative plasma ctDNA levels are predictive of PFS at 3 years. PFS at 3 years in this population is estimated at 45%. Assuming that 50% of patients will have postoperative plasma ctDNA greater than or equal to 0.5 ng/mL (1‐2 weeks after surgery) and considering a 2-year inclusion period, an α risk of 5%, a power of 80%, and a 10% censoring rate (lost to follow-up or non-evaluable at 3 years), recruiting 60 patients will allow detection of a hazard ratio of 2.38 [3,4]. This assumption was based on published feasibility studies showing high preoperative ctDNA detection rates and persistent postoperative ctDNA detection in patients who subsequently developed recurrence, although no previous prospective study has established an exact prevalence estimate for postoperative ctDNA positivity in this population. Therefore, a conservative estimate of 50% was selected for sample size calculation [16,17].

### Statistical Analysis

#### Primary Objective

PFS will be estimated using the Kaplan-Meier method and compared using the log-rank test. Postoperative plasma ctDNA as a predictive factor for PFS will be analyzed using univariate and multivariate Cox proportional hazards models. Bivariate analyses will identify covariates associated with PFS; variables with *P*<.1 will be included in the multivariate Cox model. Results will be presented as adjusted hazard ratios with 95% CIs. The predictive threshold will be determined by the ctDNA concentration maximizing the hazard ratio.

#### Secondary Objectives

The same analysis will be applied to pretreatment and posttreatment ctDNA levels (surgery±radiotherapy±chemotherapy) as predictors of tumor control (clinical recurrence). A time-dependent survival model will evaluate the effect of ctDNA evolution during follow-up on PFS and overall survival, considering quantitative ctDNA as a time-dependent variable in the Cox model. Disease-specific survival will be analyzed using the Fine and Gray model to account for competing risks from noncancer-related death.

Patient characteristics will be described and compared between those with and without progression. The Student *t* test will be used for normally distributed continuous variables; otherwise, the Mann-Whitney test will be applied. Chi-square tests will compare qualitative variables. Multivariate logistic regression will identify discriminative parameters between patients with and without progression, and the area under the receiver operating characteristic curve will be calculated.

A random-effects ANOVA (mixed model) will analyze longitudinal changes in quantitative ctDNA levels among patients with and without progression, with time as the fixed effect and patient as the random effect.

Relative and absolute changes in ctDNA concentrations preoperatively and postoperatively will be described. The association between ctDNA variation and tumor size will be analyzed using Spearman or Pearson correlation coefficients, depending on data distribution. Comparisons of ctDNA changes across resection margin categories (R0, R1, and R2) will be conducted using ANOVA if data are normally distributed or a Wilcoxon-Mann-Whitney test with Benjamini-Hochberg correction otherwise.

All statistical analyses will be performed using RStudio (version 2022.07.2+576; Posit Software, PBC). A significance threshold of 5% will be applied.

### Study Procedures

#### Information and Consent

Before participation in minimal-risk interventional research, free, informed, and explicit consent (written or oral) will be obtained after comprehensive information is provided by the investigator during a consultation and after a sufficient reflection period. The sponsor may request the participant’s consent for the use of their data in future scientific research. Participants may withdraw consent at any time.

If protocol amendments require revisions to the information forms and new consent, the ethics committee will review the procedures for obtaining this new consent. For studies involving genomic or proteomic analyses, the form must specify the type of research. Use of samples for purposes other than those for which they were initially collected is allowed unless the participant objects after being properly informed. Exceptions to the obligation to inform may occur if the participant cannot be contacted or if the relevant ethics committee considers such information unnecessary.

Personal information will be stored in the investigator’s file, treated confidentially, and made available for review by authorized authorities. According to Article 1122-1-1, withdrawal of consent does not affect activities already carried out or the use of data obtained prior to withdrawal [28].

After a reflection period and signing of the informed consent form, and verification of the eligibility criteria, the patient will be included.

#### Inclusion Process

A user guide will be provided to investigators at site opening, detailing system access and inclusion procedures. Access will be secured by individual passwords. Upon inclusion, an automatic email notification will be sent to the investigator and sponsor (Textbox 2).

Textbox 2. **The inclusion visit for all patients.**

The inclusion visit comprises the following:

- Clinical examination, including nasofibroscopy to the larynx, full inspection of the oral cavity and oropharynx, and palpation for cervical lymphadenopathy.
- General health assessment (World Health Organization score), weight, and height.
- Medical and cancer history.
- Evaluation of functional signs: phonation, swallowing, and breathing.
- Review of complete staging: ear, nose, throat–thorax computed tomography, positron emission tomography–computed tomography, and panendoscopy under general anesthesia, if available.
- Histopathology report confirming squamous cell carcinoma of the upper aerodigestive tract (diagnostic biopsy or initial excision).
- Collection of tobacco and alcohol consumption data.
- Verification of eligibility criteria and signing of informed consent.

#### Follow-Up Visits

All visits will coincide with standard care appointments. Each patient will have up to 4 blood draws for research purposes. The first visit, or inclusion visit, comprises examinations if the patient consents. Blood samples (20 mL in 2 × 10-mL tubes) will be collected the day before or the day of surgery, followed by a second 20-mL sample within 18 hours postoperatively. Tissue samples (surgical specimen and lymph nodes) will be collected and processed according to standard procedures. The third blood draw will occur12 to 18 days postsurgery or before discharge.

Blood samples: 20 mL (2 × 10-mL tubes) collected the day before or the day of surgery, followed by a second 20-mL sample within 18 hours postoperatively.

Surveillance visits will occur every 3 months, with a window of 15 days before and 15 days after the exact date, for 24 months as part of standard follow-up.

In the CIRVAD study, patients will be followed up for up to 28 months postsurgery.

Textbox 3 describes the follow-up visits.

Textbox 3. **Follow-up visits.**

Follow up visits will comprise these steps:

- Clinical examination, including nasofibroscopy down to the trachea and complete inspection of the oral cavity and oropharynx using 2 tongue depressors and appropriate lighting, followed by intraoral palpation to detect any hard cervical lymph nodes.
- Assessment of general condition (World Health Organization performance status) and weight.
- Evaluation of functional signs, including phonation, swallowing, and breathing.
- Collection of results from ear, nose, throat–thorax computed tomography scans, if performed.
- Collection of tobacco and alcohol consumption data.

A 20 mL blood sample will be collected at the end-of-study visit.

#### Sample Collection, Handling, and Transfer

For research blood samples, each patient will have up to four 20-mL blood draws. After collection, samples will be processed by the laboratory at each participating center to prepare plasma. Plasma will be double-centrifuged according to recommendations and frozen at −80 °C until transfer to the centralized analysis laboratory [29]. Detailed procedures will be provided to the centers at study initiation. The feasibility of the study workflow will be evaluated by monitoring patient recruitment, compliance with scheduled blood collections, sample processing quality, ctDNA extraction success rate, and the proportion of evaluable plasma samples. Standardization across participating centers will be ensured through harmonized operating procedures, investigator training before study initiation, centralized laboratory analyses, and predefined quality-control criteria for sample handling and ctDNA analyses.

Upon receipt, ctDNA will be extracted from plasma using an automated method (Maxwell HT ccfDNA kit; Promega). DNA concentration will be measured by fluorometry (Qubit), and fragment size will be assessed (Fragment Analyzer; Agilent). In addition to ctDNA quantification, methylation profiling will be performed because aberrant DNA methylation represents an early and frequent molecular event in head and neck squamous cell carcinoma. Hypermethylation of genes such as CALCA, ALX4, and HOXD9 [30] has previously been associated with tumor development and may improve the sensitivity of ctDNA detection by identifying tumor-specific epigenetic alterations. Methylation profiling will then be performed: enzymatic conversion will target specific methylation sites relevant in head and neck cancers, particularly CALCA (calcitonin-related polypeptide α), ALX4, and HOXD9 genes. Healthy controls will consist of plasma samples collected from healthy volunteers and processed under identical laboratory conditions. These samples will serve as nonmethylated controls for quality assessment and interpretation of methylation analyses.

For future analyses, tissue samples used for the initial diagnosis of squamous cell carcinoma of the upper aerodigestive tract will be requested from participating centers. These will be formalin-fixed, paraffin-embedded samples with greater than or equal to 10% tumor cellularity. The sponsor will arrange the centralized transfer of tissue samples at the end of the study via a dedicated carrier, in compliance with applicable regulations. Upon receipt, DNA will be extracted and stored at −80 °C, after which the tissue samples will be returned to the participating centers.

Residual plasma and extracted DNA from blood samples, as well as tissue DNA extracts, will be stored to constitute a biobank. This collection will enable future scientific research in oncology. Samples and associated data may be shared with national or international research teams or private laboratories within the European Union. An information sheet and consent form will be provided to patients at study initiation to obtain consent for participation in the biobank, including the storage of plasma, ctDNA, residual unused blood sample material, and tumor DNA extracted from tissue samples collected during standard care. The biobank will be stored at the centralized analysis laboratory.

#### Criteria for Study Termination

The study may be suspended or terminated by the sponsor in consultation with the coordinator, or at the request of the competent authority and/or ethics committee, once the study has been submitted to and authorized by the competent authority and/or ethics committee, for the following reasons: insufficient patient recruitment, poor quality of data collection, repeated major protocol violations, or publication of new scientific data undermining the rationale for this study.

#### Premature Withdrawal From the Study

Participants may leave the study prematurely for the following reasons: withdrawal of consent—participants may withdraw consent at any time, for any reason, without justification and without losing the right to receive standard care; patient death; and disease progression.

#### Special Cases

Patients will remain in the protocol and blood samples will continue to be collected for up to 28 months postsurgery, according to their consent, if they experience severe toxicity requiring permanent discontinuation of treatment; experience toxicity requiring delay or dose reduction of treatment; or refuse to continue treatment.

Refuse to continue treatment, will remain in the protocol, and blood samples will continue to be collected for up to 28 months postsurgery, according to their consent.

Figure 1 describes the study timeline. Table 1 summarizes the investigations carried out during the study.

**Figure 1.**
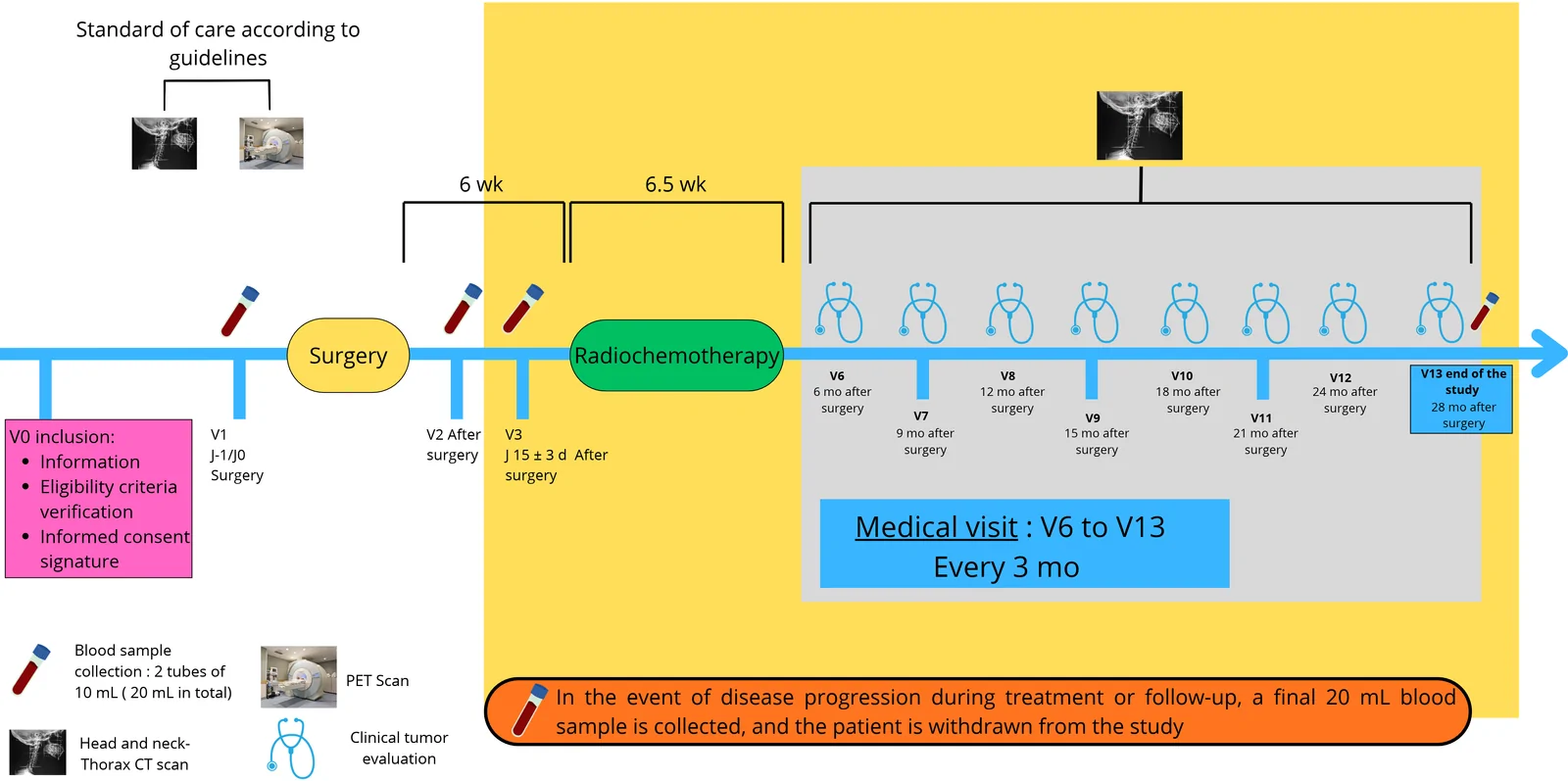
Study timeline. CT: computed tomography; PET: positron emission tomography.

**Table 1.** Summary of study investigations.

Procedures performed or data collected	Information and inclusion visit (visit 0)	Visit 1 (day before or day of surgery prior to the procedure)	Visit 2 (intraoperative)	Visit after surgery (visit 3; 15 days [±3 d] after surgery or before patient discharge)	Follow-up visit and end-of-study visit (visits 6-13; every 3 mo ± 15 d)	Disease progression visit (if progression)
Procedures performed or data collected as part of standard care						
Nasopharyngoscopy	✓				✓	✓
Oral cavity and oropharynx inspection and intraoral palpation	✓				✓	✓
WHO^a^ performance status, weight, and height^b^	✓				✓	✓
Medical history of cancer	✓					
Assessment of functional signs, with evaluation of phonation, swallowing, and respiration	✓				✓	✓
Tobacco and alcohol consumption	✓					
Histological results	✓				✓	✓
PET^c^ scan	✓					
Head and neck–thorax CT^d^ scan	✓				✓^e^	✓^e^
Panendoscopy of the upper aerodigestive tract	✓					
Biopsy	✓					
Procedures performed or data collected specifically for this research						
Patient information and consent signature	✓					
Eligibility criteria verification	✓					
Blood sample		✓	✓	✓	✓	✓

a WHO: World Health Organization.

b Height data collected only at the inclusion visit.

c PET: positron emission tomography.

d CT: computed tomography.

e If realized.

## Results

As of August 2026, study preparation, including protocol development and methodological validation, has been completed. The study is currently entering the implementation phase, with regulatory procedures, funding applications, and site initiation activities underway. No participants had been recruited at the time of manuscript submission, as recruitment has not yet begun. Recruitment is expected to start following regulatory approval and funding acquisition. The funding status remains pending, with funding applications underway as of August 2026. Participant recruitment and data collection are projected to take approximately 24 months, followed by a 28-month follow-up period. Data analysis has not yet started and will be conducted after completion of the follow-up period. The first study results are therefore expected to become available after the completion of data collection and follow-up, although a precise publication timeline (season/year) cannot yet be determined.

## Discussion

### Principal Findings

The CIRVAD study was designed to address one of the major unmet clinical needs in HPV-negative head and neck squamous cell carcinoma, namely, the lack of reliable biomarkers capable of detecting minimal residual disease and predicting recurrence before clinical or radiological evidence becomes apparent. Based on previous studies suggesting the potential value of ctDNA monitoring, we hypothesize that postoperative and longitudinal ctDNA assessment may improve risk stratification and enable earlier identification of patients at high risk of relapse.

To address this need, the CIRVAD study was designed as a prospective bicentric study including a homogeneous population of treatment-naïve patients with HPV-negative, nonmetastatic head and neck squamous cell carcinoma treated by primary surgery. Compared with previous studies, which were generally limited by small sample sizes, heterogeneous patient populations, mixed HPV status, or different treatment strategies, this study aims to provide more robust prospective evidence regarding the clinical utility of longitudinal ctDNA monitoring.

### Strengths of the Study

Several methodological strengths support the design of the CIRVAD study. First, the prospective design minimizes selection and information biases compared with retrospective studies. Second, the bicentric recruitment strategy should improve the external validity of the findings. Third, serial blood sampling at predefined time points allows evaluation of ctDNA kinetics throughout patient follow-up rather than relying on a single measurement. Finally, standardized laboratory procedures and a predefined statistical analysis plan are expected to improve the reproducibility and robustness of the study.

### Limitations

This study also presents several anticipated limitations. First, as a pilot study including 60 patients, the statistical power may remain limited for some secondary analyses and subgroup comparisons. Second, although the study population has been restricted to HPV-negative patients treated by surgery, adjuvant treatment strategies may differ according to pathological findings. To reduce the impact of this heterogeneity, treatment modalities will be incorporated into multivariable statistical models whenever appropriate. Finally, as this study is conducted in 2 French tertiary centers, external validation in larger multicenter and international cohorts will be required before widespread clinical implementation.

### Futures Perspectives

If ctDNA monitoring demonstrates sufficient predictive performance, future interventional studies could evaluate ctDNA-guided surveillance strategies and personalized treatment adaptation. Such approaches may include intensified imaging follow-up, earlier salvage surgery, or treatment escalation in patients presenting with molecular evidence of residual disease before clinical recurrence. Moreover, future protocol amendments could consider extending this strategy to patients receiving neoadjuvant immunotherapy, considering recent therapeutic advances in head and neck cancer.

Longitudinal ctDNA monitoring in patients with HPV-negative head and neck squamous cell carcinoma. By evaluating postoperative molecular residual disease and its association with clinical outcomes, this study may contribute to the development of more personalized surveillance strategies and earlier therapeutic interventions. If the proposed biomarker is validated, ctDNA monitoring could become a valuable complement to conventional postoperative follow-up and support the implementation of precision oncology in head and neck cancer.

Dissemination of the study results will include presentations at national and international scientific meetings and publication in peer-reviewed journals. The findings will also contribute to the development of future multicenter studies evaluating ctDNA-guided management strategies in head and neck cancer.

## Abbreviations

CNIL: National Data Privacy Commission
CPP: Comité de Protection des Personnes
ctDNA: circulating tumor DNA
ENT: ear, nose, throat
HPV: human papillomavirus
PFS: progression-free survival
SPIRIT: Standard Protocol Items: Recommendations for Interventional Trials
